# Does Obesity Aggravate Climacteric Symptoms in Postmenopausal Women?

**DOI:** 10.1055/s-0042-1745789

**Published:** 2022-07-12

**Authors:** Juliene Gonçalves Costa, Raquel Moreira Rodrigues, Guilherme Morais Puga, Nádia Carla Cheik

**Affiliations:** 1 Laboratory of Cardiorespiratory and Metabolic Physiology, Faculdade de Educação Física e Fisioterapia, Universidade Federal de Uberlândia, Uberlândia, Minas Gerais, MG, Brazil

**Keywords:** climacteric symptoms, obesity, overweight, menopause, sintomas climatéricos, obesidade, sobrepeso, menopausa

## Abstract

**Objective** To determine if there is a correlation between body mass index (BMI) and climacteric symptoms in postmenopausal women.

**Methods** The study sample was composed of 109 postmenopausal women with a mean age of 57 ±  8 years, mean body mass index (BMI) of 30 ±  6 kg/m ^2^ , and 8 ±  8 years after menopause. For the assessment of the climacteric symptoms, the Blatt-Kupperman Index (BKI), the Menopause Rating Scale (MRS), and the Cervantes Scale (CS) were used. Data analysis was performed through the Chi-squared test, analysis of variance (ANOVA) with the Bonferroni post hoc test, and multiple linear regression. The level of significance adopted was of *p* < 0.05. The statistical analyses were performed using the Statistical Package for the Social Sciences (IBM SPSS Statistics for Windows, IBM Corp., Armonk, NY, United States) software, version 26.0.

**Results** The multiple linear regression showed a positive association ( *p* < 0.01) between BMI values and menopause symptoms when adjusted for age and time after menopause in the 3 questionnaires used (BKI: B = 0.432; CS: B = 304; and MRS: B = 302). Regarding symptom scores, the obese women had higher mean scores ( *p* < 0.05) when compared to eutrophic women (BKI = 28 ±  10 and 20 ±  10; and MRS = 20 ±  10 and 13 ±  7, respectively). In the Chi-squared analysis, 28% of obese women had severe symptoms and 46% had moderate symptoms, while only 1% and 46% of eutrophic women had these same symptoms.

**Conclusion** There is an association between BMI and climacteric symptoms, and overweight or obese women have more intense and moderate symptoms than eutrophic women.

## Resumo

**Objetivo** Verificar se há correlação entre o índice de massa corporal e os sintomas do climatério em mulheres na pós-menopausa.

**Métodos** Participaram do estudo 109 mulheres na pós-menopausa, com idade média de 57 ±  8 anos, índice de massa corporal (IMC) médio de 30 ±  6 kg/m ^2^ e 8 ±  8 anos após a menopausa. Para a avaliação dos sintomas climatéricos, foram utilizados os questionários específicos para essa população: Índice de Kupperman-Blatt (IKB), Menopause Rating Scale (MRS), e Escala de Cervantes (EC). A análise dos dados foi realizada por meio do teste do chi-quadrado, análise de variância ( *analysis of variance* , ANOVA, em inglês) com o teste *post hoc* de Bonferroni e regressão linear múltipla. O nível de significância adotado foi *p* < 0,05. Todas as análises estatísticas foram realizadas no programa Statistical Package for the Social Sciences (IBM SPSS Statistics for Windows, IBM Corp., Armonk, NY, Estados Unidos), versão 26.0.

**Resultados** A regressão linear múltipla mostrou associação positiva ( *p* < 0,01) entre os valores do IMC e os sintomas do climatério quando ajustados pela idade e pelo tempo após a menopausa nos 3 questionários utilizados (IKB: B = 0,432; CE: B = 304; e MRS: B = 302). Quanto às pontuações dos sintomas, as mulheres com obesidade apresentaram médias maiores ( *p* < 0,05) quando comparadas às mulheres eutróficas (IKB = 28 ±  10 e 20 ± 10; e MRS = 20 ± 10 e 13 ± 7). Na análise pelo chi-quadrado 28% das mulheres obesas apresentaram sintomas graves, e 46%, moderados, ao passo que apenas 1% e 46% das eutrópicas apresentavam esses mesmos sintomas.

**Conclusão** Há uma associação entre IMC e sintomas climatéricos, e mulheres com sobrepeso ou obesidade apresentam sintomas mais intensos e moderados do que mulheres eutróficas.

## Introduction

With the increase in the life expectancy of women in Brazil, the fact that menopause occurs at the approximate age of 50 years results in a greater number of women living almost one-third of their lives in postmenopause. 1

The onset of symptoms is related to the tissues and organs that have estrogen receptors (α and β), and, in a situation of hypoestrogenism, clinical manifestations are present, with the most common being those related to genitourinary syndrome of menopause (GSM), osteoporosis, and vasomotor symptoms. Hot flashes, night sweats, and palpitations are vasomotor symptoms that affect 80% of women during the climacteric period, with 50% experiencing effects on quality of life due to the intensity and frequency of these symptoms. 2 3

The symptoms are due to multiple factors, which, in addition to low levels of estrogen, are related to the aging process and to psychosocial factors, and, according to several longitudinal population studies, 4 5 ethnic, geographic, and individual factors also affect the prevalence and severity of symptoms. Therefore, not all changes (such as those regarding libido, mood, cognition, or weight gain) are specific to menopause, although they may be secondary to vasomotor symptoms and subsequent insomnia. Several diseases associated with menopause, such as obesity, coronary vascular disease, cancer of the reproductive organs, depression, and dementia may also occur. 3

In addition to changes in the reproductive system, a lifestyle with increased physical inactivity, added to the changes resulting from the aging process, leads to important changes in the body composition of women during this period, with a gradual reduction in metabolism and increases in body and fat mass, which may reach 0.7 kg per year. 6 7 8 9

Physiological changes during the climacteric period occur not only due to ovarian failure, but also due to changes in hypothalamic and pituitary functioning. 10 In addition, estrogen is a hormone that directly effects fat tissue, the inflammatory profile, and oxidative stress. 11 These changes resulting from the climacteric, specifically in postmenopause period, may represent a link between obesity and the climacteric period, implying an increase and distribution of body fat and possibly affecting the frequency and intensity of symptoms.

Saccomani et al. 12 analyzed the relationship between obesity and climacteric symptoms and found a positive relationship using the Menopause Rating Scale, however, all climacteric women were considered as a population, with no stratification according to menopause stage. In a study by Essa and Mahmoud, 13 obese women were found to be 2.11 times more at risk of developing symptoms when compared to women with a normal body mass index (BMI); however, the authors assessed other correlated factors, such as chronic diseases and smoking, and analyzed the symptoms with instruments different from those used in the present study. The objective of the present study is to understand the relationship between the body mass index factor and climateric symptoms, using three different questionnaires in a group of women of the same post-menopausal climacteric stage.

Our hypothesis is that body mass (more specifically, BMI) affects the expression of climacteric symptoms, whose intensity and frequency in postmenopausal women will increase as the BMI increases. Therefore, the present study attempts to correlate BMI with climacteric symptoms in postmenopausal women.

## Methods

The sample was composed of 109 postmenopausal women recruited through advertisements in traditional media (newspapers, radio and TV) and electronic media (social networks), with telephone contact available for those interested. After contacting the women and verifying if they met the inclusion criteria, interviews were scheduled to apply the questionnaires. Women aged between 50 and 70 years were included; the other inclusion criteria were amenorrhea for at least 12 months, no current use of hormone therapy or phytoestrogens, and non-smoking status.

The present is an observational, cross-sectional study conducted from March 2018 to March 2019 through interviews. Specific questionnaires were applied to assess the climacteric symptoms and anamnesis, including data on age, time after menopause (years), and weight and height (self-reported) in order to calculate the BMI. All assessments were previously scheduled, and the questionnaires were administered at the Laboratory of Cardiorespiratory and Metabolic Physiology (Laboratório de Fisiologia Cardiorrespiratória e Metabólica, LAFICAM, in Portuguese) of the School of Physical Education and Physical Therapy (Faculdade de Educação Física e Fisioterapia, FAEFI, in Portuguese) of Universidade Federal de Uberlândia (UFU), in the State of Minas Gerais, Brazil. The study was approved by the local Ethics Committee (CAEE: 12453719.1.0000.5152).

The climacteric symptoms were assessed using the version validated for Brazilian Portuguese of the Menopause Rating Scale (MRS) and the Cervantes Scale (CS). 14 15 The Blatt-Kupperman Index (BKI), although not validated for the Brazilian population, showed reliability, 16 and has been used in other studies 17 18 19 with this population.

The BKI consists of eleven symptoms (vasomotor, paresthesia, insomnia, nervousness, melancholy, vertigo, weakness, arthralgia/myalgia, headache, palpitations, and tingling), to which different scores are attributed. The total score is classified as light (≤ 19), moderate (from 20 to 35) or intense (≥ 36). 20

The MRS consists of eleven questions that address psychological, somatic and urogenital domains. There are five possible answers for each question according to the intensity of the symptoms: none (0 points), mild (1 point), moderate (2 points), severe (3 points) and extremely severe (4 points). The total score can vary from zero to 44 points, and is classified as asymptomatic or scarce (0–4 points), mild (5–8 points), moderate (9–15 points) or severe (> 16 points). 15

The CS is a questionnaire composed of 31 questions divided into 4 domains: menopause and health (15 items), the psychic domain (9 items), couple relationships (3 items) and sexuality (4 items). For each question, there are 6 possible answers with scores from 0 to 5. In addition to a negative scale, the positive questions (4, 8, 13, 15, 20, 22, 26 and 30) have an inverted score for the total sum. The sum of the points can vary from 0 to 155, with 0 corresponding to the best quality of life in the climacteric period, and 155, the worst. 14

The sample size was calculated according to the formula presented by Tabachnick and Fidell, 21 which considers the number of explanatory variables to be included in the model (n = 50 + 8m [m is the number of explanatory variables]; given that m = 3 in the present study, a minimum of 74 women should be recruited, and we recruited 109). To verify the association between BMI and climacteric symptoms, multiple linear regression was used, with climacteric symptoms as a dependent variable (total scores of the BKI, CS and MRS), and the BMI values, adjusted for years after menopause and age, as an independent variable.

A subanalysis was performed to verify the differences between the BMI classifications (eutrophic [n = 24], overweight [n = 39], and obese [n = 46]) in relation to the questionnaire classifications (MRS and BKI). One-way analysis of variance (ANOVA) with the Bonferroni post hoc test was performed. To verify the frequency of the symptoms, the Chi-squared test was used, and, to analyze the relationship between the questionnaires, the Spearman correlation was used. The level of significance adopted was *p* < 0.05. All analyses were performed using The Statistical Package for the Social Sciences (IBM SPS Statistics for Windows, IBM Corp., Armonk, New York, United States) software, version 26.0.

## Results

The clinical data and the data regarding the symptoms are shown in Table 1 . The volunteers (n = 109) were divided into 3 groups according to the BMI, as classified by the World Health Organization (WHO): 22 eutrophic (n = 24), overweight (n = 39), and obese (n = 46).There were no differences in terms of mean age (52 ±  7, 55 ±  9, and 57 ±  5 years respectively) or mean time since amenorrhea (4 ±  7, 6 ±  8 and 5 ±  8 years respectively) among the groups. The three questionnaires used assess the climacteric symptoms, and showed significant ( *p* < 0.01), positive, and strong correlations (r > 0.7). Regarding the questionnaire scores, the eutrophic group showed fewer climacteric symptoms when compared to the obese group in the BKI ( *p* < 0.01) and MRS ( *p* = 0.01), but not in the CS (p = 0.07). There were no significant differences regarding the scores of the overweight women in any of the questionnaires when compared to the eutrophic or obese women.

**Table 1:** Clinical characteristics and questionnaire scores according to the classification of the Body Mass Index (n = 109)

**Clinical characteristics**	**Eutrophic** **(n = 24)**	**Overweight** **(n = 39)**	**Obese** **(n = 46)**
Age (years)	52 ± 7	55 ± 9	57 ± 5
Time after menopause (years)	4 ± 7	6 ± 8	5 ± 8
Body mass (kg)	58 ± 6	69 ± 5	90 ± 12
Body Mass Index (kg/m ^2^ )	22 ± 1	27 ± 1	34 ± 4
**Score on the questionnaires**			
Blatt-Kupperman Index	20 ± 10*	24 ± 8	28 ± 10
Cervantes Scale	44 ± 25	57 ± 23	58 ± 25
Menopause Rating Scale	13 ± 7*	18 ± 7	20 ± 10

Notes: One-way analysis of variance with the Bonferroni post hoc test; * *p* < 0.05: eutrophic versus obese.

Table 2 shows the frequency according to the classifications of each questionnaire and the BMI. For a better understanding of the analysis of the distribution among the questionnaires, in the MRS, which contains four classifications (asymptomatic, mild, moderate and severe), we grouped the asymptomatic and mild classifications as one. Since the CS has no classification, we chose not to present the distributions in order not to change the understanding of its score. The mild symptoms were significantly more frequent ( *p* < 0.01) in eutrophic (50%) than overweight (26%) and obese (26%) subjects, and more severe in obese (28%) than overweight (5%) and eutrophic (4%) subjects, according to the BKI.

**Table 2:** Distribution of eutrophic, overweight and obese Women according to climacteric symptoms (n = 109)

	**Eutrophic** **(n = 24)**		**Overweight (n = 39)**		**Obese** **(n = 46)**		
	**n**	**%**	**n**	**%**	**n**	**%**	***p***
*Blatt-Kupperman Index*							
Mild	12	50	10	26	12	26	< 0.01*
Moderate	11	46	27	69	21	46	
Severe	1	4	2	5	13	28	
*Menopause Rating Scale*							
Asymptomatic + mild	7	29	3	8	6	13	
Moderate	7	29	13	33	10	22	0.11
Severe	10	42	23	59	30	65	

Note: *Chi-squared p < 0.05.

Multiple linear regression was used to verify whether the proposed models (including the dependent variables: BMI, age, and time after menopause) were able to predict changes in the symptoms in the three questionnaires used. The results revealed that the models predict the severity of climacteric symptoms in 18%, 11% and 9% of the variations (r ^2^ ) of the BKI, CS and MRS respectively, and showed a moderate correlation (r values between 0.3 and 0.6). Table 3 shows the regression results for the BKI. The BMI, even when adjusted for age and time after menopause, showed a significant association ( *p* < 0.01) and greater impact on symptoms (Beta = 0.432).

**Table 3:** Significance of the multiple regression parameters of the Blatt-Kupperman Index variable

	**Unstandardized coefficients**		**Standardized coefficients**	**t**	**Siginificance**
	**B**	**Standard error**	**Beta**		
(Constant)	19.545	9.045		2.161	0.033
Body Mass Index	0.723	0.150	0.432	4.817	0.000
Age	-0.310	0.170	-0.237	-1.829	0.070
Time after menopause	0.199	0.159	0.160	1.250	0.214

*t value:* test for the statistical significance of each of the independent variables.

Table 4 shows the regression results for the CS. Again, the BMI, even when adjusted for age and time after menopause, showed a significant association ( *p* < 0.01) and greater impact symptoms (Beta = 0.304).

**Table 4:** Significance of the multiple regression parameters of the Cervantes Scale variable

	**Unstandardized coefficients**		**Standardized coefficients**	**t**	**Significance**
	**B**	**Standard error**	**Beta**		
(Constant)	68.949	23.614		2.920	0.004
Body Mass Index	1.271	0.392	0.304	3.243	0.002
Age	-0.999	0.443	-0.305	-2.255	0.026
Time after menopause	0.613	0.415	0.197	1.478	0.142

*t value:* test for the statistical significance of each of the independent variables.

Table 5 shows the regression results for the MRS. Once more, the BMI, even when adjusted for age and time after menopause, showed a significant association ( *p* < 0.01) and greater impact symptoms (Beta = 0.302).

**Table 5:** Significance of the multiple regression parameters of the Menopause Rating Scale variable

	**Unstandardized coefficients**		**Standardized coefficients**	**t**	**Significance**
	**B**	**Standard error**	**Beta**		
(Constant)	8.838	8.176		1.081	0.282
Body Mass Index	0.432	0.136	0.302	3.184	0.002
Age	-0.075	0.153	-0.067	-0.486	0.628
Time after menopause	0.070	0.144	0.066	0.489	0.626

*t value:* test for the statistical significance of each of the independent variables.

## Discussion

The present study was performed to verify the association between menopausal symptoms and body mass in postmenopausal women. The results showed that the frequency and intensity of symptoms increase as the BMI increases, with differences regarding eutrophic, overweight and obese statuses, relating that the change in classification according to body mass is already sufficient to affect the quality of life during the climacteric period.

Climacteric symptoms are the most common clinical manifestations during this period, and they are due to multiple factors, which, in addition to low levels of estrogen, are also related to the aging process and psychosocial factors. These symptoms affect approximately 80% of women, and 50% of this population undergoes impacts on quality of life due to their intensity and frequency. 3 23 Therefore, tools that enable a complete analysis of these symptoms are necessary in order to recognize them. For this, we used three different but complementary questionnaires, which made it possible to understand not only the symptoms (MRS), but also the quality of life related to the climacteric period (CS) and based on the outpatient routine (BKI).

Saccomani et al. 12 used the MRS and found an average score of 10, but in a group of Brazilian women who were younger (with ages ranging from 45 to 60 years) and in another climacteric stage, which would justify the lower score obtained by them when compared to the mean scores of 17 found in the present study. In line with our findings, Ruan et al. 24 observed higher frequency and severity of symptoms among postmenopausal women (aged between 50 and 70 years) than among women in earlier climacteric stages, such as perimenopause. In addition, there may be an association between the onset of long-term symptoms and more visible effects of aging, such as, atrophy and vaginal dryness, thus generating more symptoms and, consequently, higher scores. 25

In the study by Yim et al., 26 the variables of age, obesity and level of physical activity demonstrated an association with the severity of symptoms. The age factor seems to mainly affect sexual and vasomotor symptoms, while the obesity factor seems to be more related to physical and vasomotor symptoms. In the present study, we did not assess the level of physical activity, but studies 17 27 have shown that active women have fewer symptoms than inactive women, and that after 10 weeks of moderate physical exercise, the climacteric symptoms can be reduced by up to 50%.

The results of the present study demonstrate that obesity, as well as overweight status, are important and significant factors in the increase in climacteric symptoms; and we found that, the higher the BMI, the higher the frequency and intensity of the symptoms. Adipose tissue, even in small amounts, can be pathogenic due to the adverse consequences of excessive fat mass and/or negative endocrinological activity, and it is associated with many metabolic diseases. 28 In a study involving Spanish women conducted by Fernández-Alonso et al., 29 the presence of metabolic diseases seemed to be an aggravating factor for climacteric symptoms, as well as age and overweight status.

Considering the average increase of 0.7 kg/year in weight during the climacteric period 9 and the growing projection of overweight women (from 29% to 53.9%) and obesity (8% to 20.7%) based on data from the last 35 years, attention, treatment and monitoring of this population is necessary since the first signs of uncontrolled body mass or overweight are observed, so that the condition does not worsen, as stated by Pereira and Lima 30 in a study with Brazilian women.

Postmenopausal women are at a higher risk of developing cancer and cardiometabolic diseases, and one of the influencing factors, in addition to lifestyle parameters (such as physical inactivity, unhealthy eating habits), is the reduction in the levels of estrogen, which is characteristic of menopause that can cause changes in energy expenditure and reduced leptin sensitivity. 31 Estrogen is involved in the regulation of several physiological processes, and has receptors (α and β) in different organs and tissues. This activity affects the inflammatory profile, oxidative stress, acts directly on adipose tissue, and, more specifically, controls energy balance, adiposity and distribution of body fat, as well as the onset of climacteric symptoms. 10 11

Excess adiposity is considered an important factor that affects women's quality of life during menopause, and it has been associated with longer duration of menopause, higher incidence of general symptoms and higher prevalence of vasomotor symptoms. 4 Obesity is related to autonomic imbalance, with greater chronic sympathetic activation and reduced parasympathetic activation, which could increase the presence of symptoms in this population. In addition, changes in thermoregulation and reduction in heat release occur due to the accumulation of adipose tissue. 32 33

Individual factors associated with obesity, such as low schooling and economic levels, poor general health and stressful events, also favor the onset and worsening of symptoms. 4 34 Data from several longitudinal studies have shown that the prevalence of symptoms can also vary among countries due to ethnic, regional and climatic factors, such as increased or reduced exposure to sunlight. Consistent with this, African women appear to be affected by more persistent symptoms, European and Latin American women experience a higher prevalence of hot flashes and insomnia, while Scandinavian women are at an increased risk of suffering osteoporotic fractures, and women from warmer countries have a higher incidence of hot flashes and night sweats. 4 35

One of the main limitations of the present study is its small sample size, with different numbers of participants in each BMI subgroup. We did not assess the level of physical activity or socioeconomic aspects, since this was not the objective of the present work, but this might have complemented the results obtained and broadened the understanding of the influencing factors.

One of the strengths of the study is that we used three specific questionnaires that allowed a reliable evaluation of the data, with results that complement each other. In addition, another strong point is the stratification of the sample based on the climacteric stage, more specifically in the post-menopausal period. Since the stages of climacterium may present different patterns in the frequency and intensity of symptoms due to hormonal fluctuations, homogenizing the sample according to the same period after menopause makes it possible to understand the influence of obesity specifically for this population, under hormonal conditions as similar as possible.

## Conclusion

Climacteric symptoms are related to BMI, and, in postmenopausal women, the gradual increase in BMI in turn increases the frequency and severity of the symptoms. Understanding this relationship can contribute to the development of public policies for the care of women during the climacteric.
